# Retraction Note: Knockdown of FOXO3a induces epithelialmesenchymal transition and promotes metastasis of pancreatic ductal adenocarcinoma by activation of the βcatenin/TCF4 pathway through SPRY2

**DOI:** 10.1186/s13046-022-02413-2

**Published:** 2022-06-17

**Authors:** Jun Li, Rumeng Yang, Yuting Dong, Manyao Chen, Yu Wang, Guoping Wang

**Affiliations:** 1 Institute of Pathology, Tongji Hospital, Tongji Medical College, Huazhong University of Science and Technology, 1095 Jiefang Dadao, Wuhan, 430030 People’s Republic of China; 2 Department of Pathology, School of Basic Medicine, Tongji Medical College, Huazhong University of Science and Technology, Wuhan, 430030 People’s Republic of China

**Retraction Note: J Exp Clin Cancer Res 38, 38 (2019)**

**https://doi.org/10.1186/s13046-019-1046-x**

The Editor-in-Chief has retracted this article. A Correction was published in 2021 [1]. Subsequently concerns have been raised with respect to both the original figures and the figures in the Correction. The authors have not provided a satisfactory response to these concerns and so the Editor-in-Chief no longer has confidence in the data presented. All authors disagree with this retraction.
